# Novel and Sensitive Touchdown Polymerase Chain Reaction Assays for the Detection of Goat and Sheep Milk Adulteration with Cow Milk

**DOI:** 10.3390/molecules29081820

**Published:** 2024-04-17

**Authors:** Ariadni Kourkouli, Nikolaos Thomaidis, Marilena Dasenaki, Athina Markou

**Affiliations:** 1 Laboratory of Analytical Chemistry, Department of Chemistry, National and Kapodistrian University of Athens, 15771 Athens, Greece; arkourko@chem.uoa.gr (A.K.); ntho@chem.uoa.gr (N.T.); 2 Laboratory of Food Chemistry, Department of Chemistry, National and Kapodistrian University of Athens, 15771 Athens, Greece

**Keywords:** milk adulteration, bovine, goat, sheep, PCR, touchdown PCR, assays

## Abstract

Milk is the most consumed liquid food in the world due to its high nutritional value and relatively low cost, characteristics that make it vulnerable to adulteration. One of the most common types of milk adulteration involves the undeclared addition of cow’s milk to milk from other mammalian species, such as goats, sheep, buffalo or donkeys. The incidence of such adulteration not only causes a crisis in terms of commercial market and consumer uncertainty but also poses a risk to public health, as allergies can be triggered by proteins in undeclared cow’s milk. In this study, a specific qualitative touchdown (TD) PCR method was developed to detect the undeclared addition of cow’s milk in goat and sheep milk based on the discrimination of the peak areas of the melting curves after the modification of bovine-specific primers. The developed methodology has high specificity for the DNA templates of other species, such as buffalos and donkeys, and is able to identify the presence of cow’s milk down to 1%. Repeatability was tested at low bovine concentrations of 5% and 1% and resulted in %RSD values of 1.53–2.04 for the goat–cow assay and 2.49–7.16 for the sheep–cow assay, respectively. The application of this method to commercial goat milk samples indicated a high percentage of noncompliance in terms of labeling (50%), while a comparison of the results to rapid immunochromatographic and ELISA kits validated the excellent sensitivity and applicability of the proposed PCR methodology that was able to trace more adulterated samples. The developed assays offer the advantage of multiple detection in a single run, resulting in a cost- and time-efficient method. Future studies will focus on the applicability of these assays in dairy products such as cheese and yogurt.

## 1. Introduction

According to the FAO, liquid milk is the most consumed dairy product in developing countries due to its high nutritional value. The demand for milk and dairy products is continuously increasing due to rising incomes, population growth, urbanization, and modern dietary trends. However, according to FAO’s Dairy Market Review (2022), dry and warm weather combined with high input costs, especially for fuel, fertilizer and feed, influenced by the ongoing Russian–Ukrainian war, are contributing to a future decline in European dairy production [1].

These conditions create an ideal environment for increased milk fraud. According to a 2013 European Parliament report, milk is one of the four ingredients/foods most frequently targeted for economically motivated adulteration. In fact, many fraud cases go undetected, so the number of cases reported through the Rapid Alert System for Food and Feed (RASFF) or through the media is underestimated compared to the actual number [2]. Commercial adulteration includes mixing expensive, high-quality milk such as buffalo, sheep, and goat milk with cow milk, which is cheaper and seasonally available [3]. Assessing the authenticity of dairy products is important not only from an economic perspective but also because of consumers’ medical requirements (e.g., allergies to certain milk proteins), religious practices, or other personal choices [4]. Another important issue is the protection of the characteristics and reputation of traditional cheeses with Protected Designation of Origin (PDO) and Protected Geographical Indication (PGI), as described in Commission Regulation (EC) 1151/2012, which sets requirements for milk from certain species and/or in certain quantities [5,6,7].

In an effort to overcome these problems and gain consumer confidence, laws have been enacted throughout the world. A variety of analytical methods have been proposed to comply with these regulations. As described in a review by Mafra, Honrado and Amaral [8], these methods are mainly based on proteins or lipids and include electrophoresis, immunochemistry, chromatography and mass spectrometry. The major drawbacks of all these techniques are their relatively low sensitivity and their unsuitability for heat-treated material or for discrimination between closely related materials. This fact led to the rapid development of molecular methods because DNA is highly persistent during food processing and can retain retrievable sequence-specific information after an amplification reaction (PCR) [9].

DNA-based applications for the authentication of milk and dairy products have been extensively discussed in recent years [8,10,11,12,13,14] due to the fact that the PCR method with DNA as a target can still show good sensitivity, even after heat-treating dairy products [15]. Several methods have been described, including DNA sequencing with restriction fragment length polymorphism (RFLP) [16,17] and random amplified polymorphic DNA (RAPD) [18], as well as next-generation sequencing (NGS) [19], but PCR technology remains the main method for DNA detection. Most PCR tests described are based on classical PCR in combination with gel electrophoresis [20,21,22], which does not allow for the quantification of the target. In addition, quantitative real-time PCR assays have been developed using either SYBR Green followed by melting temperature ™ analysis and high-resolution melting PCR (HRM) [23,24] or TaqMan probes for the simultaneous detection of more than one target [25,26,27]. Finally, assays using absolute quantification technologies such as droplet digital PCR (ddPCR) have been performed to a limited extent [28]. However, due to the high similarity of DNA sequences in several species, such as sheep, cows and goats, as well as buffalos and cattle, the specificity of the primers and probes used in PCR tests is controversial.

In the present study, a sensitive and specific methodology was developed for the detection of different DNA targets to indicate the adulteration of sheep or goat milk by the addition of cow milk simultaneously in a single reaction. Due to the high homology between the three species, a much more specific and sensitive touchdown PCR (TD-PCR) protocol was developed that aims to avoid misclassification and the formation of non-specific products. This method also has the potential to overcome problems with the high annealing temperatures required for some primer-template combinations and is particularly useful for difficult-to-amplify templates, such as those with extensive secondary structures or high %GC content [29]. The performance of hybridization and the overall success of the PCR method were evaluated using melting analysis.

## 2. Results and Discussion

### 2.1. Assay Optimization

To optimize the assays for cow–sheep or cow–goat detection, DNA extracted from raw cow, sheep, and goat milk was used. Ruminant milk can be easily used as a DNA source because it contains a large number of somatic cells, mainly leukocytes, but also epithelial cells of the milking mother, which contain genomic DNA suitable for PCR amplification [30]. In the milk of healthy cows, the cell concentration ranges from 10^7^ to 10^4^ cells/mL and is highly dependent on clinical status [31].

**Figure 1:**
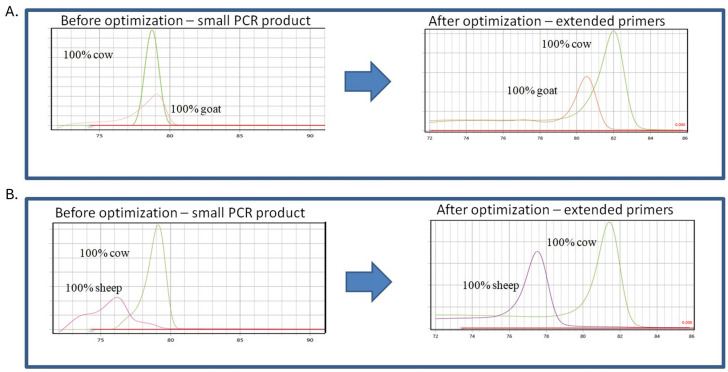
The figure shows assay optimization: temperature of melting peaks obtained using qPCR assay using specific primers, (**A**) cow–goat assays before and after optimization, (**B**) cow–sheep assays before and after optimization.

In all cases, the final conditions were selected based on the best differentiation of the melting curve analysis. In addition, PCR protocols were optimized according to the final concentration of magnesium, BSA, primer concentration and TMAC addition. Due to the presence of non-specific signals, further optimization was performed with a different number of PCR cycles and a different temperature program (Touch Down PCR). The effects of PCR product size were also investigated, having found in initial experiments that the signals obtained for cow and goat DNA had the same Tm. It was hypothesized that by increasing the length of the cow target sequence, very good discrimination between the different milk species would be achieved. Therefore, two different bovine-specific primer pairs were designed and tested; one pair amplified short sequences and the other pair amplified longer sequences with additional extensions (Table 1). It was found that the larger the cow PCR product, the stronger the discrimination between the peaks of the melting curve analysis (Figure 1). Each species was confirmed via melting curve analysis: the pure samples showed melting curves with a single inflection point at Tm values of 82 °C, 80.5 °C and 77 °C for cows, goats and sheep, respectively (Figure 1A,B).

### 2.2. Analytical Validation

Analytical validation of the developed assays was performed using synthetic DNAs and/or DNA from raw milk samples to estimate the specificity, limit of detection (LOD), intra-assay repeatability and analytical recovery.

#### 2.2.1. Specificity

**Figure 2:**
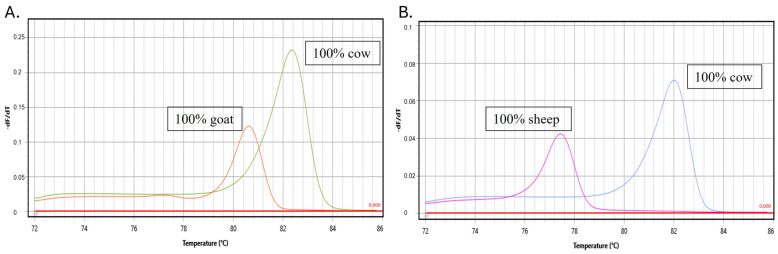
The figure shows the analytical specificity of the developed assays using raw milk from cows, sheep, goats, donkeys and buffalos for (**A**) cow–goat assay and (**B**) cow–sheep assay.

Synthetic reference DNA templates of each species and authentic raw milk were used to evaluate the analytical specificity of the assays. The specificity of each specific primer was checked for the presence or absence of nonspecific amplifications using the temperature of the melting peak (°C). The primers developed for each species were specific and showed no non-specific amplifications compared to the raw milk samples of the other species or synthetic DNA controls (Figure 2). Donkey and buffalo milk samples were also compared, and no non-specific amplifications were observed. Each specific primer had a specific peak melting temperature.

#### 2.2.2. Sensitivity

**Figure 3:**
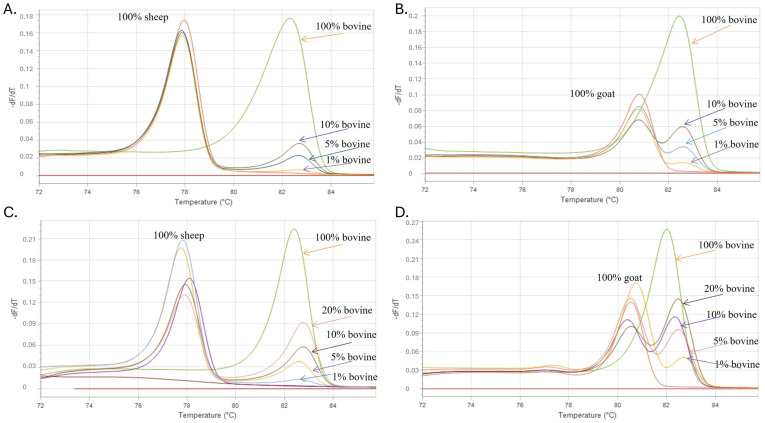
The figure shows melting curves analysis of both assays showing sensitivity of cow–sheep assay both in synthetic controls and raw samples (**A**,**C**) and of cow–goat assay both in synthetic controls and raw samples (**B**,**D**).

To assess the sensitivity of the proposed methodology, adulterated cow and sheep samples were prepared in the laboratory at different ratios: 1, 5, 10, 20 and 50% (*v*/*v*) cow milk was mixed with 99, 95, 90, 80 and 50% (*v*/*v*) goat or sheep milk to produce binary milk mixtures. DNA isolated from these mixtures was analyzed using the developed methodology. In addition, a synthetic DNA control of each species was used to simulate the adulteration of goat or sheep DNA and verify the authentication ability of the developed assay. The results obtained with the developed doublex TD-PCR methods showed that the method could clearly detect the incorporated cow milk components in goat (Figure 3B,D) or sheep milk (Figure 3A,C), and the minimum LOD was 1% (*v*/*v*). The sensitivity of the developed assays is satisfactory since, according to Commission Regulation (EC) 273/2008, fraud in milk is defined when a value is equal to or higher than 1% [32]. In most cases, the detection of milk adulteration in percentages of <1% could indicate unintentional contamination due to poor handling rather than economically motivated adulteration.

#### 2.2.3. Intra-Assay Repeatability

For the evaluation of the intra-assay repeatability of the developed assay, biased simulations were analyzed by mixing bovine milk with goat or sheep milk at ratios of 1% and 5% in triplicate. SD and CV% were calculated according to the guidelines for minimum information for the publication of quantitative real-time PCR experiments [33]. Intra-assay repeatability was assessed by analyzing triplicate samples within the same analytical run (Table 2). The CV% for all cases was <10%, verifying the accuracy of the developed assay.

Serial dilutions of synthetic controls of each species were used for the evaluation of the PCR efficiency of each assay [34]. The amplified target DNA for both assays had at least 90% efficiency; specifically, 98.84% efficiency (slope: −3.45) was achieved for the cow–goat assay and 95.6% (slope: −3.43) was achieved for the cow–sheep assay.

#### 2.2.4. Recovery–Quality Control

The recovery of the methods was determined using spiked-in DNA-EC in known concentrations for each individual reaction. The recovery rate of DNA-EC was estimated in each case from the amount of an equivalent number of DNA-EC copies that we had added to the eluted DNA after the extraction step (corresponding to 100% recovery). The recoveries of all the simulated samples were between 84% and 103%, confirming the accuracy of the developed assays.

### 2.3. Application of the Developed Assay in Commercial Goat Milks

**Figure 4:**
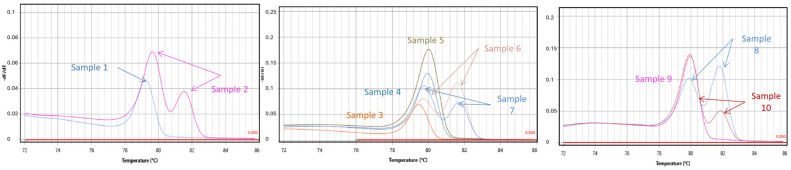
The figure shows the application of the developed assay in milk: melting curves analysis of cow–goat assays in commercial milk samples labeled as 100% goat milk.

The developed doublex TD-PCR methods were used to analyze 10 commercial goat milk samples, all from different brands available on the Greek market. All of the analyses were performed in duplicate. The results of this test were extremely interesting: five out of ten samples (samples 1, 3, 4, 5 and 9) showed only one positive peak in Tm, verifying the presence of goat milk, indicating that these five samples are pure goat milk containing no cow milk. However, melting curve analysis of the other five samples (2, 6, 7, 8 and 10) showed an additional positive amplification in the Tm of the bovine PCR product, suggesting that these five samples were adulterated with cow’s milk. The double peaks of the above samples demonstrated the amplification of both bovine and goat milk components (Figure 4).

Using real-time PCR assays and TaqMan probes, Tsakali et al. (2019) previously showed that 90% of commercial goat milk and dairy products in the Greek market were adulterated with cow’s milk [7]. These results, combined with the results of our study showing a high proportion of cow’s milk adulteration, are disappointing when considering the honesty of food labeling. Di Pinto et al. (2017) tested the authenticity of 80 goat cheeses from the Italian market and found that 80% were adulterated with cow/sheep milk [35]. The evaluation of these results shows that there is a high percentage of non-compliance with labeling requirements, indicating either insufficient technology and knowledge in milk collection and processing or economically motivated adulteration of goat milk, which is considered a high-quality product with excellent nutritional properties. Whether intentional or unintentional, the effects of mislabeling dairy products include consumer deception and potential health risks. Our research verifies that there is a tremendous need for increased inspections of importers, retailers and distributors to reduce dairy mislabeling and detect food fraud in a robust and efficient manner.

### 2.4. Direct Comparison Study between the Developed Assay, ELISA and Rapid Immunochromatographic Test

To verify the results of the developed methodology, two commercial kits for the detection of bovine DNA were used: the first was based on the ELISA immunoenzymatic reaction (RC bovino kit), while the second consisted of an immunochromatographic strip test (IC bovino kit). Both kits were used according to the manufacturer’s instructions (Zeulab S.L., Zaragoza, Spain). The developed methodology and the two commercial kits were used to analyze the same samples in order to allow a direct comparison of the results. DNA extracted from the ten commercial goat milks and two additional milk blends of 1% bovine–99% goat and 5% bovine–95% goat milk, respectively, were used. 

Using the developed assay, cow’s milk was detected in five out of ten commercially available goat’s milks, while 5% and 1% mixed milks were also correctly detected. Using the ELISA method, cow’s milk was detected in only two out of ten commercial goat’s milks while the 5% and 1% mixed milks were incorrectly detected as 100% goat’s milk (false negative). The concordance between the developed assay and the ELISA was quite good at 7/10 (70%) (Table 3). More specifically, five samples were found to be negative when using both methods, while three samples were found to be positive only with the developed TD-PCR assay. When the results were analyzed performing the Cohen’s kappa coefficient (κ) test, it was found that there was a “fair” agreement between these two methods that was not statistically significant (κ = 0.400, Table 3).

A similar comparative study was performed between the use of an immunochromatographic rapid test and the developed TD-PCR assay. Using the rapid test, cow’s milk was detected in only two out of ten commercial goat’s milks, while the 5% and 1% mixed milks were falsely identified as authentic goat milk. The agreement between the developed TD-PCR assay and rapid test was higher than ELISA, with 8/10 (80%) (Table 3). More specifically, five samples were found to be negative with both methods, while two samples were found to be positive only with the developed assay. When the results were analyzed by performing the Cohen’s kappa coefficient (κ) test, it was found that there was a “moderate” agreement between these two methods that was not statistically significant (κ = 0.600, Table 3).

Our results are in contrast to those of López-Calleja et al. [36], who indicated that both ELISA and PCR can be specific and reliable tools for the detection of low levels of undeclared cow’s milk in sheep’s and/or goat’s milk cheese and other dairy products. This discrepancy can be explained by the different target molecules and the principles and sensitivity levels of the individual methods. However, it is important to emphasize that there are very few studies on the direct comparison of milk samples through the use of PCR, ELISA and other established methods used in daily practice.

## 3. Materials and Methods

### 3.1. Sample Collection

Synthetic reference DNA templates (Integrated DNA Technologies (IDT), Coralville, IA, USA) of the mitochondrial gene of cytochrome c oxidase subunit I (cox1DNA) of each species were used to develop and validate the cow–goat and cow–sheep assays. In addition, to test method applicability, 8 authentic cow, 11 goat and 6 sheep milk samples were provided by local farmers. To increase specificity, one donkey and one buffalo milk sample were obtained and analyzed using the developed methodology. In addition, binary mixtures with decreasing cow DNA concentrations of 50, 20, 10, 5 and 1% (*v*/*v*) were prepared. A synthetic control (DNA) was used as an external control to estimate the amount of DNA lost during the extraction protocol (%Recovery). Finally, 10 commercial milk samples labeled as 100% goat milk were obtained from the Greek market and analyzed to verify their animal origin.

### 3.2. DNA Extraction and Recovery Evaluation

#### 3.2.1. DNA Extraction

DNA was extracted from the milk samples using the DNEasy Blood and Tissue Kit (Qiagen, Hilden, Germany). First, 1 mL of milk was centrifuged at 6000 rpm/15 min, and the supernatant was discarded. The remaining cell pellet was treated according to the manufacturer’s instructions (spin-column protocol for non-nucleated mammalian blood). Briefly, 20 μL of proteinase-K and 200 μL of PBS were added to the pellet and incubated for 2 h at room temperature. Then, 200 μL of lysis buffer (AL) was added and incubated at 56 °C/10 min. Following this, 10 μL of 10^4^ (copies/μL) cfDNA was spiked into the solution as an external control. After the addition of 200 μL of ethanol (100%), the solution was pipetted into the mini-spin columns, each time with the appropriate wash buffer. Finally, DNA was recovered after the addition of 100 μL of elution buffer (AE), and its concentration was determined using the Nanodrop ND-ONE spectrophotometer (Thermo Scientific, Waltham, MA, USA). The DNA extracts were stored at −20 °C until use. 

#### 3.2.2. Evaluation of DNA Recovery

The concentration and purity of the DNA were determined through the use of absorbance measurements at 260 and 280 nm using the Nanodrop ND-ONE spectrophotometer. In addition, the external DNA control added in the first step of the extraction procedure was amplified with specific primers via PCR to verify its presence and evaluate the recovery of the whole process in all isolated DNAs (Table 1). The PCR reactions of the external DNA were performed under the following thermal cycling conditions: an initial denaturation at 95 °C for 2 min; 95 °C for 10 s for 40 cycles, 61 °C annealing for 15 s and a final extension at 72 °C for 15 s. Finally, melting analysis was performed with a reduction of 0.1 °C/s within a temperature range of 95–55 °C. All of the amplification reactions were performed in a mixture (10 µL) containing 0.2 µL dNTP mixture (10 mM), 2 µL of PCR buffer (5×), 1.2 µL of MgCl2 (25 mM), 0.15 µL of BSA (10 μg/μL), 0.3 µL of each primer (10 µM), 1 µL of LC Green dye, 0.1 µL of Taq polymerase (5 U/μL) and 1 µL of template DNA and brought to a final reaction volume of 10 µL with DNase/RNase-free water.

### 3.3. Design of Species-Specific Primers

The primer design was based on the mitochondrial cytochrome c gene as it has been shown to be a suitable molecular marker for the classification and identification of closely related animal species [37]. Based on Giglioti et al. (2022) [3], primers for cox1DNA of the mitochondrial gene (Genbank ΜΖ668303, ΜΖ782619 and ΜΖ782720 for bovine, goat and sheep, respectively) were designed de novo in silico, synthesized via IDT and evaluated for performance (Table 1). Primer Premier 5.0 software (Premier Biosoft International, San Francisco, CA, USA) was used to avoid primer dimer formation, false priming sites, hairpin structure formation and homology with other genes. All of the primers were designed to fit the assay conditions, such as amplicon sizes and melting temperatures. The specificities of all primers were first tested via homology searches using BLAST (Basic Local Alignment Search Tool—NCBI).

To perform perfect Tm differentiation in the PCR product calculated via melting analysis, specific extensions were added to the primer pair for bovines. Specifically, the bovine upstream primer consisted of a stem–loop extension of 43 bases (5′-GAAAGAAGGCGAGGACGGAAGAATGTGCGTCTCGCCTTCTTTC-3′) and approximately 21 nucleotides (nt) of gene-specific sequence, whereas the bovine downstream primer consisted of approximately 22 nt of a gene-specific sequence and a 10-base extension (5′-ATTCATTATC-3′) at the 5′ end.

The specificity and universality of the primers were verified by using specific primers and reference primers for amplification and in authentic raw goat, cow and sheep milk provided by local farmers.

### 3.4. Duplex TD-PCR Protocols

Duplex touchdown PCR (TD-PCR) was performed with 1 µL of DNA in a final volume of 10 µL. A PCR-negative control containing no target was included in each test run. The reaction consisted of 1 µL of PCR buffer (5×) (Promega, Madison, WI, USA), 2 µL of MgCl_2_ (25 mM), 0.2 µL of dNTP mix (10 mM), 0.5 µL of (10 μg/µL) BSA, 0.6 µL of 0.5 M TMAC (tetramethylammonium chloride), 1 µL of LC Green fluorescent dye and 0.1 µL of Taq polymerase (5 U/ µL). After the primer concentrations were optimized, 0.2 μΜ of the bovine-specific primers and 0.5 μM of the sheep-specific primers were set for the cow–sheep assay, while equal amounts of 0.6 μΜ of both primers were preferred for the cow–goat assay. The samples were cycled in the MIC PCR cycler (Biomolecular Systems, Upper Coomera, Queensland, Australia). The final PCR conditions were as follows: initial denaturation at 95 °C for 3 min, followed by 16 cycles of 95 °C for 20 s, 67 °C (with a reduction of 1 °C for each successive cycle), annealing for 30 s, 72 °C for 30 s, followed by 20 cycles of 95 °C for 20 s, 58 °C annealing for 30 s and a final extension at 72 °C for 30 s. Finally, melting analysis was again performed with a reduction of 0.1 °C/s within a temperature range of 90–72 °C. Two positive synthetic controls for each specific species were used in each experiment, and the peak (T m) of each control determined which species it was.

## 4. Conclusions

In conclusion, the development and application of two novel TD-PCR assays developed for the detection of goat’s and sheep’s milk adulteration with cow’s milk was presented. Compared to typical ELISA and immunochromatographic rapid tests, these assays represent highly sensitive, rapid and efficient alternative methods that can be routinely used to control milk authenticity. The developed assays offer the advantage of multiple detection in a single run, resulting in a cost- and time-efficient method. Future studies will focus on the applicability of these assays in dairy products such as cheese and yogurt. This study underlines the importance of food traceability and quality control using specific and sensitive analytical methods and clearly demonstrates the need for the implementation of effective and accurate monitoring and tracking programs to ensure effective species identification in dairy products. Enforcing the guidelines of European legislation would require the development and application of reliable traceability systems to ensure the efficiency of food safety systems. In addition, continuous monitoring combined with improved detection methods and strict penalties for defaulters may help minimize authentication issues in the future.

## Figures and Tables

**Table 1:** The table shows sequences of the primers that were used in the developed douplex TD-PCR assays of this study.

	Sequence (cox1 Gene)	Product Length (bp)	Product T_m_ (°C)
**Bovine (*Bos taurus*)**			
Forward (5′→3′)	TTAATCTTACCTGGGTTTGGA	120	79
Reverse (5′→3′)	GAAACCTAGAAATCCGATTGAC		
**Extended primers bovine**			
Loop Forward (5′→3′)	GAAAGAAGGCGAGGACGGAAGAATGTGCGTCTCGCCTTCTTTCTTAATCTTACCTGGGTTTGGA	173	82
Extended Reverse (5′→3′)	ATTCATTATCATTCATTATCGAAACCTAGAAATCCGATTGAC		
**Goat (*Capra hircus*)**			
Forward (5′→3′)	CTTATTTTACCTGGATTTGGA	124	80.5
Reverse (5′→3′)	CAATAAATCCTAGAAACCCGA		
**Sheep (*Ovis aries*)**			
Forward (5′→3′)	TTTGGGATAATCTCCCATATT	93	77
Reverse (5′→3′)	CCCAATTGATATTATGGCTCAT		
**External Control (synthetic control)**			
Forward (5′→3′)	TGTTAGCAACTCTTCAAGTTCCCT	128	86
Reverse (5′→3′)	AGGCAGGTAGGGCTGGAACA		

**Table 2:** The table shows intra-assay repeatability: the cycle threshold (Ct) values of the douplex TD assays.

	Cq_1_	Cq_2_	Cq_3_	Mean Cq	SD	%RSD
1% cow–99% sheep	8.15	9.11	7.68	8.31	0.60	7.16%
5% cow–95% sheep	9.88	9.84	9.35	9.69	0.24	2.49%
1% cow–99% goat	8.54	8.22	8.15	8.30	0.17	2.04%
5% cow–95% goat	8.41	8.66	8.71	8.59	0.13	1.53%

**Table 3:** Comparison between the developed TD-PCR cow-goat assay and (a) ELISA and (b) rapid immunochromatographic sticks for 10 commercial goat milks.

		**Sticks (IC bovino)**		Total
		0	1	
**TD-PCR** **Cow-goat assay**	Negative	5	0	5
	Adultarated samples	2	3	5
K-cohen = 0.60				
		**ELISA**		Total
		0	1	
**TD-PCR** **Cow-goat assay**	Negative	5	0	5
	Adultarated samples	3	2	5
K-cohen = 0.40				

## Data Availability

Data are available upon request.
